# Tools for Antigen Delivery: From Traditional Nanocarriers and Biomimetic Platforms to Emerging Physical, Bioengineered and Computational Approaches

**DOI:** 10.3390/vaccines14060516

**Published:** 2026-06-09

**Authors:** Liying Sun, Yujiao Miao, Deyun Jiang, Chao Liu

**Affiliations:** 1 NHC Key Laboratory of Systems Biology of Pathogens, Key Laboratory of Pathogen Infection Prevention and Control (Ministry of Education), State Key Laboratory of Respiratory Health and Multimorbidity, National Institute of Pathogen Biology, Chinese Academy of Medical Sciences & Peking Union Medical College, Beijing 100005, China; miaoyujiao@ipbcams.ac.cn (Y.M.); jiangdeyun@ipbcams.ac.cn (D.J.); 2 State Key Laboratory of Vaccines for Infectious Diseases, Xiang An Biomedicine Laboratory, and Fujian Provincial Key Laboratory of Innovative Drug Target Research, School of Pharmaceutical Sciences, Xiamen University, Xiamen 361102, China; 3 Shenzhen Research Institute of Xiamen University, Shenzhen 518000, China

**Keywords:** antigen presentation, drug delivery systems, nanocarriers, biomimetic platforms, cross–presentation, cancer vaccine

## Abstract

The magnitude and quality of adaptive immune responses are fundamentally influenced by the efficiency of antigen presentation. Traditional vaccine platforms, such as live–attenuated or inactivated pathogens, although immunogenic, often present safety concerns. Conversely, subunit vaccines, despite being safer, generally exhibit poor immunogenicity due to inadequate delivery of antigens to professional antigen–presenting cells (APCs). To address this issue, the development of innovative delivery systems has become a pivotal strategy to overcome significant biological barriers, including extracellular antigen degradation, suboptimal lymph node targeting, and inefficient cross–presentation necessary for CD8+ T cell activation. This review systematically explores recent advancements in delivery technologies aimed at enhancing antigen presentation, encompassing rationally engineered nanocarriers and sophisticated biomimetic platforms. We first examine how nanoparticle properties like size, surface charge, and ligand density affect intracellular trafficking and the transition from MHC–II to MHC–I cross–presentation. Then, we explore bioinspired systems such as extracellular vesicles, virus–like particles, and cell–membrane–coated nanoparticles that utilize natural biological traits for enhanced targeting and immune modulation. Additionally, we review new physical delivery methods like microneedle arrays and in situ electroporation for direct, minimally invasive antigen delivery to dendritic cells. Lastly, we discuss the potential of these platforms in personalized cancer vaccines and combination immunotherapies. By combining insights from materials science, immunology, and bioengineering, these next–generation delivery tools could enhance antigen presentation and transform precision vaccination and immune intervention.

## 1. Introduction

Enhanced antigen presentation constitutes a fundamental strategy in the advancement of next–generation vaccines and immunotherapies, with the objective of delivering antigens efficiently and precisely to antigen–presenting cells to provoke a robust adaptive immune response [1,2]. In recent years, the intersection of materials science, immunology, and bioengineering has facilitated the development of three key categories of innovative delivery tools [3]. The first category includes synthetic nanocarriers, such as lipid nanoparticles, polymeric nanoparticles, and inorganic nanoparticles, which offer advantages in the precise regulation of their physicochemical properties and scalability in production [4]. The second category encompasses biomimetic and cell–derived platforms, including exosomes, virus–like particles, and cell membrane–coated nanoparticles; these platforms leverage naturally evolved biological interfaces to provide unique benefits in active targeting and immune modulation [5]. The third category encompasses physical delivery mechanisms, such as microneedle arrays and nano–electroporation [6,7]. By enabling precise spatial delivery, targeted delivery to specific anatomical sites such as skin, lymph nodes, or tumor tissues, and application of physical forces, such as electric fields or microneedle–mediated disruption to facilitate cellular uptake and endosomal escape, these tools effectively complement the first two categories in clinical translation. This review systematically explores the design principles, immunological mechanisms, and progress in clinical translation associated with these three categories of delivery tools, focusing on their role in enhancing antigen presentation. The objective is to identify common challenges and propose future directions for development.

In addition to enhancing antigen presentation efficiency, antigen delivery systems also critically determine the polarization direction of innate and adaptive immune responses, as reflected by the induction of distinct helper T cell subsets such as Th1, Th2, and Th17. This polarization process is closely linked to the antigen presentation pathway, specifically whether the antigen is presented via the endogenous MHC class I pathway or the exogenous MHC class II pathway. Generally, the MHC class I pathway primarily activates CD8+ T cells and favors Th1 type immune responses, which are suitable for antiviral and antitumor immunity. In contrast, the MHC class II pathway mainly activates CD4+ T cells, which can further polarize into Th1, Th2, or Th17 subsets depending on microenvironmental signals. Therefore, an ideal antigen delivery system should not only focus on the efficiency of antigen presentation but also consider how it directs the polarization of immune responses. Different delivery platforms, such as lipid nanoparticles, polymeric carriers, or biomimetic platforms, exhibit significant differences in intracellular delivery, endosomal escape, and antigen processing pathway selection, which ultimately shape the resulting immune response profile. Accordingly, this review also aims to systematically compare various delivery platforms and elucidate their effects on CD4+ and CD8+ T cells.

Different delivery sites require distinct techniques and approaches. At the cellular level, delivery methods are mainly categorized into three tiers: extracellular delivery, endosomal delivery, and cytosolic delivery. Extracellular delivery releases antigens into the extracellular matrix or interstitial space, relying on uptake by antigen–presenting cells (APCs); its advantage is simplicity and low demands on carrier design, but efficiency is limited by cellular phagocytic capacity. Endosomal or lysosomal delivery is the default pathway for most nanocarriers after endocytosis, where antigens are confined within endosomal compartments, processed, and primarily loaded onto MHC class II molecules to activate CD4+ T cells. Cytosolic delivery requires carriers to possess endosomal escape capability, allowing antigens to penetrate the endosomal membrane into the cytosol, where they are processed by proteasomes and presented via MHC class I molecules to activate CD8+ T cells; common strategies include pH–responsive membrane fusion and membrane perforation. At the tissue level, delivery approaches depend on the lesion type and its microenvironmental characteristics. For solid tumors, delivery strategies primarily utilize the enhanced permeability and retention (EPR) effect for passive targeting or surface–modified ligands for active targeting, while also needing to overcome barriers such as dense extracellular matrix and high interstitial pressure. For inflammatory sites and injured areas, increased local vascular permeability and immune cell infiltration allow delivery systems to accumulate passively, with further targeting of infiltrating inflammatory cells or APCs. For lymphoid organs, delivery systems require appropriate sizes to drain via lymphatic vessels to lymph nodes, directly acting on resident dendritic cells and lymphocytes.

## 2. Synthetic Nanocarriers

### 2.1. Lipid Nanoparticles (LNPs)

**Figure 1:**
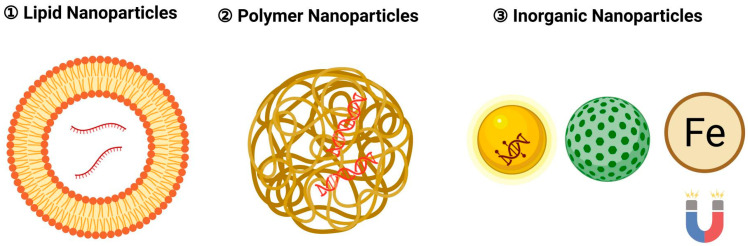
Representative examples of nanoparticle platforms for antigen delivery. Shown are common types, including lipid nanoparticles, polymeric nanoparticles, inorganic nanoparticles, exosomes, virus–like particles, and cell membrane–coated nanoparticles. These are representative examples and do not encompass all possible nanoparticle types. The numbers ①–③ in the figure indicate the three categories of synthetic nanocarriers: ① Lipid Nanoparticles, ② Polymer Nanoparticles, and ③ Inorganic Nanoparticles. Colors are used solely to distinguish different nanoparticle types and carry no additional meaning.

Lipid nanoparticles are a nanoscale delivery system formed through the self–assembly of components such as ionizable lipids, auxiliary lipids, cholesterol, and polyethylene glycol–conjugated lipids [8] (Table 1). Auxiliary lipids, also known as helper lipids, are typically phospholipids that stabilize the lipid bilayer, modulate membrane fluidity, and promote endosomal escape. These lipid vesicles can efficiently encapsulate and protect nucleic acid therapeutics such as mRNA, siRNA, and antisense oligonucleotides. Among these components, ionizable lipids play a key role in the immune evasion and cross–delivery capabilities of LNPs. At physiological pH (7.4), ionizable lipids maintain a neutral charge, reducing nonspecific protein adsorption and serum clearance while prolonging circulation time in vivo. Upon endocytosis into cells, the acidified environment of the endosomal lumen (pH 5.0–6.0) triggers the protonation of the ionizable lipids, causing them to acquire a positive charge and disrupt the endosomal membrane via electrostatic interactions, thereby facilitating the cytoplasmic release of antigens such as mRNA [9,10]. Gong et al. designed a cross–linked ionizable lipid, C12–2aN, which not only promotes endosomal escape to enhance mRNA expression but also stimulates glycolysis in dendritic cells by activating the mTORC2 pathway, demonstrating potent vaccine efficacy in both SARS–CoV–2 and tumor vaccine models [11]. Among auxiliary lipids, unsaturated phospholipids (such as DOPE) tend to form hexagonal phase structures to facilitate fusion with the endosomal membrane and aid in endosomal escape, whereas saturated phospholipids enhance antigen presentation and co–stimulatory molecule expression in dendritic cells. Hussain et al. systematically compared the effects of different auxiliary lipids (DSPC, DOPC, DOPE) and sterols (cholesterol, β–sitosterol) on LNP performance, finding that the DSPC/cholesterol combination achieved the highest luciferase expression following intramuscular injection, while DOPE–containing LNPs enhanced total IgG and IgG1 antibody responses [12]. As a membrane modulator, cholesterol regulates membrane fluidity and rigidity by inserting into the phospholipid bilayer, thereby enhancing the structural stability of LNPs; its derivatives may also modulate the intensity of immune responses. Hussain et al. also found that replacing cholesterol with β–sitosterol could induce higher levels of cytokines such as TNF–α and IL–6 [12]. Pegylated lipids form a hydration layer that reduces plasma protein adsorption via steric hindrance, enabling LNPs to evade the immune system, though their proportion must be optimized to avoid accelerated blood clearance [13] (Table 2). By synergistically regulating the stability, in vivo distribution, cellular uptake, and endosomal escape efficiency of LNPs, these auxiliary lipids collectively balance the immune evasion and cross–multifunctional presentation functions of LNPs (Figure 1).

In addition, Park et al. developed DEC–205 antibody–functionalized LNPs (dLNPs), which, by targeting the DEC–205 receptor highly expressed on the surface of dendritic cells, significantly enhanced antigen presentation by MHC class I molecules and elicited a robust antitumor T–cell immune response [21,22]. Wang et al. developed H–type ionizable lipid–based LNPs (H18NPs). By enriching the surface protein crown with complement C3 protein, these LNPs achieved specific transfection of splenic dendritic cells. Their splenic DC transfection efficiency was 1.84 times that of commercially available MC3–LNPs, and they achieved an inhibition rate as high as 95.9% in the B16–OVA tumor model [23]. These studies establish that rational design and functional modification of LNP components enable synergistic regulation of immune evasion and cross–presentation, providing a crucial foundation for the development of next–generation nucleic acid vaccines and immunotherapies.

### 2.2. Polymer Nanoparticles

Polymer nanocarriers are nanoscale delivery systems constructed from natural or synthetic polymeric materials through various fabrication methods, including self–assembly, emulsification, nanoprecipitation, microfluidics, and spray drying. Their biocompatibility and biodegradability vary significantly depending on the polymer type; therefore, careful selection is required for vaccine delivery applications [14].

Natural polymer nanocarriers offer unique advantages in vaccine delivery due to their widespread availability and inherent biological activity. However, it is important to recognize that natural biopolymers such as proteins and glycoproteins possess intrinsic immunogenic and antigenic properties, which may either benefit or interfere with vaccine–induced immune responses depending on the context. Among these, chitosan, a linear cationic polysaccharide, is rich in amino groups along its molecular chain, enabling it to efficiently load negatively charged antigens and oligonucleotide adjuvants through electrostatic interactions [15]. Chitosan’s cationic properties confer excellent mucosal adhesion, allowing it to bind to the mucus layer via electrostatic interactions and prolong its retention time on the mucosal surface [15]. However, these cationic properties also promote the adsorption of serum proteins and opsonization, accelerating recognition and clearance by the reticuloendothelial system. After polyethylene glycol (PEG) modification, a “masked” hydration barrier forms on the surface of chitosan nanoparticles, effectively reducing nonspecific protein adsorption, decreasing priming effects and macrophage uptake, and thereby prolonging the in vivo half–life [24,25]. More importantly, chitosan itself possesses mucosal adhesion and adjuvant activity, capable of opening tight junctions between epithelial cells, facilitating the passage of nanovaccines through the mucosal barrier, and enabling their uptake by antigen–presenting cells (APCs) [26,27]. Furthermore, the protonation properties of chitosan allow it to buffer proton influx in the acidic environment of endosomes, inducing a “proton sponge effect” that disrupts the endosomal membrane, thereby facilitating the cytoplasmic release of antigens and promoting MHC class I cross–presentation [16]. Liu et al. utilized fluorocarbon–modified chitosan (FCS) to significantly enhance its hydrophobicity and transmembrane ability, enabling it to self–assemble with ovalbumin (OVA) and CpG to form an inhalable nanovaccine (FCS–OVA–CpG). This vaccine not only successfully penetrated the nasal epithelium and was phagocytosed by APCs but was also delivery to lymph nodes, simultaneously activating both humoral and cellular immunity. Subsequently, the team further leveraged the permeation–enhancing properties of FCS to develop a transdermal nanovaccine platform, enabling non–invasive delivery of antibodies and antigens, and demonstrating strong potential in melanoma immunotherapy and viral vaccines [17,28].

Synthetic polymeric nanocarriers have become a core material in cancer vaccine research due to their tunable chemical structures, high batch–to–batch reproducibility, and controllable degradation rates. As an FDA–approved biodegradable polyester, Polylactic–co–glycolic acid (PLGA) can efficiently encapsulate both hydrophilic and hydrophobic antigens and adjuvants via the double emulsion method or nanoprecipitation. PLGA can also be PEGylated (as described above for chitosan) to reduce macrophage clearance and prolong circulation, thereby enhancing passive targeting to lymph nodes or tumor tissues. Additionally, the hydrophobic core of PLGA protects the encapsulated antigen from enzymatic degradation, further maintaining its stability in vivo [29,30]. A key advantage of PLGA is its ability to enable programmed release of antigens and adjuvants. By adjusting the polymer molecular weight and the lactic acid/glycolic acid ratio, the duration of immune stimulation can be extended from weeks to months, mimicking the persistent infection signals of pathogens and thereby enhancing germinal center responses and the formation of memory T cells [18,31]. Liu et al. utilized PLGA to simultaneously encapsulate whole–cell cancer antigens (including both soluble and insoluble components) dissolved in 8 M urea along with TLR agonists. The resulting nanovaccine achieved 100% prevention of lung cancer and 70% prevention of melanoma in mice [32]. PEI is a classic cationic polymer known for its high density of amine groups and powerful “proton sponge effect”. Low–molecular–weight PEI can be modified via PEGylation or fluorination to reduce cytotoxicity, decrease serum protein adsorption, and prolong circulation [19]. At physiological pH, PEI efficiently aggregates nucleic acid antigens into nanocomposites via electrostatic interactions; upon entering cells, it undergoes extensive protonation in the acidic environment of endosomes, leading to an influx of chloride ions that increases endosomal osmotic pressure and causes rupture, thereby achieving efficient cytoplasmic delivery. However, the significant cytotoxicity of high–molecular–weight PEI limits its application; therefore, researchers often use low–molecular–weight PEI or modify it to balance safety and transfection efficiency. For example, Xu et al. simply mixed fluorinated low–molecular–weight PEI (F13–PEI) with OVA or cancer cell membrane antigens, which self–assembled to form a nanovaccine [19]. This vaccine not only significantly enhanced antigen cross–presentation and OVA–specific T–cell responses but also demonstrated good in vivo safety [33]. The aforementioned studies indicate that PLGA and PEI optimize sustained immune activation and cytoplasmic delivery efficiency, respectively, through distinct physicochemical mechanisms (controlled release and endosomal escape).

### 2.3. Inorganic Nanoparticles

Inorganic nanoparticles offer irreplaceable advantages in vaccine delivery due to their unique physicochemical properties, such as surface plasmon resonance, superparamagnetism, and mesoporous structures [20]. These materials typically feature a high specific surface area, precisely controllable morphology and size, and multifunctional integration capabilities. They can efficiently load antigens and adjuvants and, through specific physicochemical mechanisms, enhance immune evasion and cross–presentation of antigens.

Gold nanoparticles (AuNPs) have emerged as ideal carriers for combined photothermal therapy and vaccine delivery strategies due to their excellent photothermal conversion properties, ease of surface functionalization, and good biocompatibility. The surface of gold nanoparticles can be covalently conjugated with antigen peptides, Toll-like receptor agonists, and targeting molecules to construct multicomponent nanovaccines [20]. Studies have shown that the particle size of gold nanoparticle vaccines has a decisive impact on their passive targeting efficiency to lymph nodes and the intensity of the immune response: gold nanoparticles with sizes ranging from 10 to 22 nm can be efficiently drained to lymph nodes and taken up by dendritic cells, whereas particles smaller than 10 nm are prone to rapid clearance [34]. In terms of immune evasion and cross–presentation, gold nanoparticles demonstrate unique subcellular transport capabilities. Zhao et al. prepared poly–dopamine–coated gold nanoparticles with a “burdock seed” structure (AuNX–PDA). This nanovaccine mimics the morphology of pathogens through its surface spines, enhancing physical adhesion to cell membranes and facilitating internalization by dendritic cells via the phagocytosis pathway. Notably, AuNX–PDA can direct the delivery of captured tumor antigens to the endoplasmic reticulum and Golgi apparatus, bypassing the classical lysosomal degradation pathway, thereby significantly promoting antigen cross–presentation via MHC class I molecules and activating CD8+ T cell responses [35]. Furthermore, glycosylation modifications on the surface of gold nanoparticles can regulate their recognition efficiency by antigen–presenting cells; α–mannose modification promotes dendritic cell uptake, whereas sialic acid modification has the opposite effect [20].

Mesoporous silica nanoparticles (MSNs) have attracted widespread attention for the efficient co–delivery of antigens and adjuvants due to their highly ordered mesoporous structure, ultra–high specific surface area, tunable pore size, and excellent biocompatibility. The core advantage of MSNs lies in their precisely controllable pore size (typically 2–50 nm), which enables the selective loading and programmed release of antigens with different molecular weights [36]. Ting et al. adopted an “integrated” design concept, using MSNs as a platform to achieve the co–loading of antigen proteins and nucleic acid–based immunostimulants. The resulting nanovaccines promote antigen cellular uptake, sustained release in vivo, and delivery to lymph nodes, ultimately inducing an earlier, stronger, and more durable immune response. Regarding the study of cross–presentation mechanisms, researchers found that the pore size of MSNs directly determines the efficiency of antigen cross–presentation [37]. Viswanath et al. prepared MSNs with particle sizes of approximately 80 nm, with pore sizes of 7.8, 10.3, and 12.9 nm, respectively. After loading them with ovalbumin, they found that although the three types of MSNs exhibited comparable efficiency in lymph node drainage and dendritic cell uptake, dendritic cells treated with the large–pore (12.9 nm) MSNs demonstrated the highest MHC class I cross–presentation efficiency, thereby inducing the strongest antitumor immune response. The mechanism underlying this is that the larger pore size facilitates more effective escape from lysosomal degradation during intracellular transport of the antigen, thereby allowing it to enter the cytoplasm for proteasomal processing [38].

Iron oxide nanoparticles (IONPs) have demonstrated tremendous potential in the field of nanovaccines due to their superparamagnetic properties, biodegradability, and unique immunomodulatory activity. IONPs not only serve as magnetic resonance imaging (MRI) contrast agents for tracking immune cells in vivo, but have also been found in recent years to possess intrinsic adjuvant activity, capable of activating the interferon–stimulated gene (ISG) pathway and enhancing cross–presentation efficiency [39,40]. Qi et al. engineered Fe_3_O_4_@SIINFEKL–TAT nanoparticles, in which the TAT transmembrane peptide promotes antigen uptake, while the Fe_3_O_4_ core enables dual–modality tracking via both MRI and magnetic particle imaging. This study confirmed that Fe_3_O_4_ nanoparticles can be taken up via the early–late endosomal pathway and successfully escape from lysosomes within 4 h, thereby enabling antigen cross–presentation and inducing up to 90% dendritic cell maturation in vitro [41]. Furthermore, Chen et al. developed an acid–ionizable iron nanoadjuvant (PEIM) by co–assembling IONPs with the STING agonist MSA–2. This iron nanoadjuvant responds to the acidic microenvironment at lymph node drainage sites, releasing iron ions and MSA–2, wherein the iron ions synergistically enhance type I interferon production via the STING pathway, achieving a 16–fold dose–sparing effect. The antigen–specific CD8+ T–cell response induced by the PEIM@OVA nanovaccine was 55–fold higher than that induced by soluble antigens, demonstrating potent antitumor effects in melanoma and colorectal cancer models [40]. Additionally, inspired by the NOX2 enzyme, Zhang et al. constructed a composite nanosystem (OFG) consisting of a Fe–gallic acid network and ovalbumin. This system mimics the NOX2–catalyzed cascade of reactive oxygen species production, promoting the polarization of tumor–associated macrophages toward the M1 phenotype while simultaneously inhibiting the activity of lysosomal cysteine proteases. This significantly reduces antigen degradation and enhances the efficiency of antigen cross–presentation by macrophages by 4.5–fold [42].

**Figure 2:**
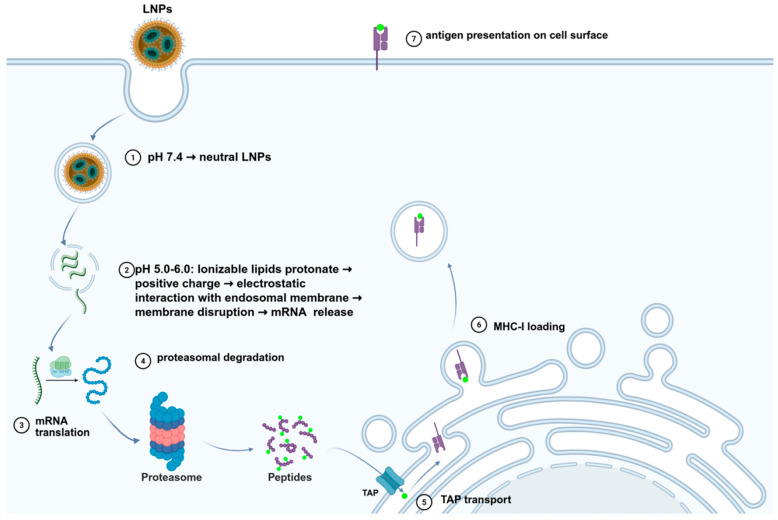
Schematic illustration of LNP–mediated cytosolic mRNA delivery and the classical MHC–I antigen presentation pathway. ① Lipid nanoparticles (LNPs) remain neutral at physiological pH (7.4) for prolonged circulation. ② Following endocytosis, ionizable lipids promote endosomal escape and cytosolic release of mRNA. ③ Ribosomes translate the delivered mRNA into antigenic protein. ④ The protein is degraded by the proteasome into short peptide fragments. ⑤ Peptides are translocated into the endoplasmic reticulum lumen via the TAP transporter. ⑥ Inside the ER, peptides are loaded onto MHC class I molecules with the assistance of chaperones. ⑦ The stable peptide–MHC–I complex is transported to the cell surface for presentation to CD8 positive cytotoxic T lymphocytes, thereby priming cellular immunity. Colors are used solely to distinguish different cellular compartments and components (LNPs, mRNA, ribosomes, proteasome) and carry no additional meaning. Arrows indicate the sequential flow of events: solid arrows denote direct progression, while the curved arrow around the endosome represents endosomal maturation. Numbers 1–7 correspond to the steps described below, following the chronological order of antigen processing and presentation.

In summary, these studies demonstrate that gold nanoparticles, mesoporous silica, and iron oxide nanoparticles, through their respective physicochemical properties (surface plasmon resonance, mesoporous confinement effects, superparamagnetism, and ion release behavior), enable lymph node–targeted delivery, antigen loading and programmed release, immune evasion, and enhanced cross–presentation, respectively. This provides a diverse range of material options for the development of highly efficient, multifunctional nanovaccine platforms (Figure 2).

## 3. Biomimetic and Cell–Derived Platforms

### 3.1. Extracellular Vesicles

Exosomes represent a predominant subtype of extracellular vesicles (EVs), characterized by a diameter of approximately 40 nanometers, and are secreted by nearly all eukaryotic cell types [43]. Recent studies have elucidated that exosomes play a pivotal regulatory role in immune communication, facilitated by their distinctive molecular composition, which includes ESCRT proteins, tetra–transmembrane proteins, Rab GTPases, and lipid mediators [44]. The biogenesis of exosomes initiates with the invagination of the plasma membrane to form early endosomes, followed by the further invagination of the endosomal membrane to generate intraluminal vesicles (ILVs) [45]. These ILVs accumulate to form multivesicular bodies (MVBs); upon fusion of MVBs with the plasma membrane, the ILVs are released into the extracellular milieu as exosomes [46]. This biogenetic pathway intersects with antigen processing and presentation pathways, with MVBs serving as critical sites for the loading of antigenic peptides onto MHC class II molecules, thereby enabling exosomes to inherently delivery MHC–antigen peptide complexes.

Exosomes possess dual immunological roles, encompassing both immunostimulatory and immunosuppressive functions, attributable to their intrinsic targeting and immunomodulatory capabilities. Exosomes originating from dendritic cells (DCs) are enriched with integrins and adhesion molecules, facilitating their efficient capture by T cells within lymph nodes [47]. Empirical evidence indicates that exosomes can activate Toll-like receptors and other pattern recognition receptors by transporting damage–associated molecular patterns (DAMPs) and pathogen–associated molecular patterns (PAMPs), thereby initiating innate immune responses through endosomal and cytoplasmic signaling pathways [48]. Notably, exosome–mediated presentation of MHC antigens occurs via both classical and non–classical pathways, with interactions between these pathways potentially modulating downstream immune signaling processes. In the context of autoimmune diseases, exosomes bearing autoantigens may present these to T cells either directly or indirectly through antigen–presenting cells, thereby impacting the maintenance or disruption of immune tolerance.

In recent years, substantial advancements have been achieved in the domain of engineered exosomes. Concerning antigen loading, a scaffold system based on HIV–1 Nefmut exploits the intrinsic propensity of the Nef protein for efficient enrichment within exosomes; by fusing the antigen to its C–terminus, a highly efficient loading process is realized [49]. The engineering of donor cells, achieved through transfection to overexpress the target antigen, ensures natural antigen processing and accurate MHC presentation. In terms of surface modification, target ligands can be presented on the surface of dendritic cell–derived exosomes to enhance specificity, while adjuvants such as Toll-like receptor (TLR) agonists and stimulator of interferon genes (STING) agonists can be incorporated to synergistically augment the immune response [50,51,52,53]. A systematic review by Adedokun et al. underscores the increasing attention being directed towards the therapeutic potential of exosomes in cancer immunotherapy and vaccine design, with their strategic integration as a next–generation immunomodulatory platform being highly anticipated [54].

### 3.2. Virus-like Particles

Virus-like particles (VLPs) are nanoparticles that form through the self–assembly of viral structural proteins, typically ranging in diameter from 20 to 200 nanometers [55,56]. A key characteristic of VLPs is the absence of viral genetic material, rendering them incapable of replication and thus considerably safer than attenuated or inactivated viruses. The structural advantages of VLPs manifest in three primary ways: firstly, their highly repetitive antigenic epitope arrays can effectively cross–link B–cell receptors, thereby eliciting a robust humoral immune response; secondly, exogenous antigens can be flexibly presented on their surface or within the particle through gene fusion or chemical conjugation; and thirdly, their nanoscale dimensions facilitate uptake by dendritic cells (DCs) and subsequent migration to lymph nodes. Furthermore, certain VLPs possess the ability to activate Toll-like receptors, thereby providing an endogenous adjuvant effect.

Recent research has significantly advanced our understanding of the mechanisms underlying immune activation induced by virus–like particles (VLPs). Published studies have elucidated the molecular processes through which B cells directly recognize VLP antigens [57]. It has been demonstrated that the repetitive presentation of surface epitopes on VLPs, known as epitope density, constitutes a distinctive biophysical characteristic that initiates B–cell receptor (BCR) signal transduction [58,59]. This repetitive pattern activates signaling pathways of varying quality and functions as an independent signal for antigen–specific B–cell activation. Variations in epitope density quantitatively modulate the extent and quality of B–cell activation, while the presence of internal nucleic acids within the particles significantly influences the persistence of the antibody response. These findings establish epitope density and internal nucleic acids as two fundamental signals for B–cell activation and antibody production, offering crucial insights for the rational design of vaccines.

Virus-like particles (VLPs) represent one of the most successful platforms in vaccine technology. Since the VLP–based hepatitis B vaccine, there has been a marked improvement in immunoprotection [60,61]. Additionally, VLP vaccines for human papillomavirus (HPV), such as Gardasil and Cervarix, are now extensively utilized for the prevention of cervical cancer [62]. In the pursuit of developing broad–spectrum vaccines, strategies involving mosaic and cocktail VLPs are being implemented to address the challenge of viral diversity. VLP vaccines targeting HIV–1, norovirus, and SARS–CoV–2 are currently in clinical trials, with some candidates advancing to Phase III studies [63]. Regarding enhanced antigen presentation, VLPs have the capability to activate CD8+ T cells through the MHC I cross–presentation pathway, thereby inducing cytotoxic T–cell responses. Ongoing research is dedicated to optimizing the size, shape, surface charge, and antigen presentation efficiency of VLPs to further augment their immunogenic properties.

### 3.3. Cell Membrane–Coated Nanoparticles

Cell membrane–coated nanoparticles (CM–NPs) represent a biomimetic platform that has gained prominence in recent years [64,65]. This innovative approach involves the encapsulation of synthetic nanoparticles with the membrane structure of natural cells, thereby imparting the synthetic carriers with the biological functionalities inherent to natural cells. The preparation process generally includes several key steps: cell lysis and collection of membrane fragments, membrane vesiculation, co–extrusion of membrane vesicles with the nanoparticle core, and subsequent characterization of the membrane coating’s integrity through techniques such as transmission electron microscopy (TEM), particle size analysis, zeta potential measurement, among others. This “top–down” strategy effectively preserves the complete protein composition of the cell membrane, including membrane proteins, glycoproteins, and lipid rafts, while simultaneously allowing for the versatile loading of antigens, adjuvants, or drugs into the core nanoparticle.

Cell membranes derived from various sources impart distinct functional specificities to nanoparticles. Specifically, red blood cell membranes facilitate prolonged circulation and immune evasion; platelet membranes enable targeting of injury sites; white blood cell membranes facilitate interactions among immune cells and promote homing to lymph nodes; dendritic cell (DC) membranes inherently express major histocompatibility complex (MHC) and co–stimulatory molecules, rendering them suitable for the development of artificial antigen–presenting cells (aAPCs); and tumor cell membranes possess a wide array of tumor–associated antigens, making them ideal for personalized tumor vaccines. Chen et al. systematically reviewed design innovations in biomimetic and personalized nanovaccines, highlighting that biomimetic platforms, such as nanoparticles coated with exosomes, erythrocyte membranes, and immune cell membranes, can enhance antigen presentation, macrophage polarization, and the initiation of adaptive immunity by reconstructing natural biological interfaces [66,67].

To enhance antigen presentation, dendritic cell (DC)–coated nanoparticles, functioning as artificial antigen–presenting cells (aAPCs), can directly activate T cells both in vitro and in vivo, bypassing the uptake and presentation processes of endogenous DCs. These nanoparticles provide signal 1 and signal 2 necessary for T cell activation. Conversely, nanoparticles coated with tumor cell membranes exploit the broad–spectrum antigens present on the tumor cell membrane to encapsulate CpG adjuvants within the nanoparticle core, thereby effectively promoting DC maturation and activating tumor–specific T–cell responses. It is noteworthy that T–cell–inspired delivery strategies, such as T–cell membrane–coated nanoparticles, T–cell–derived exosomes, and chimeric antigen receptor T (CAR–T) cells, have garnered significant attention in recent years. These approaches offer inherent biocompatibility and biodegradability, extended circulation half–lives, and the capability to traverse biological barriers.

A thorough comparative analysis of the three biomimetic platforms demonstrates that each possesses distinct advantages and limitations. Exosomes are advantageous due to their natural expression of major histocompatibility complex (MHC) and co–stimulatory molecules, their capacity to mediate cross–reactivity, and their superior biocompatibility. Nonetheless, they are hindered by low yield, complex separation and purification processes, and are predominantly in the clinical trial phase. Virus-like particles (VLPs) exhibit the most advanced clinical translation pathway, with several products having been commercially available for many years, and their highly repetitive epitopes are capable of eliciting robust B–cell responses. However, they encounter challenges such as potential carrier–specific immunity, which may limit reusability, and low assembly efficiency in certain VLPs. Cell membrane–coated nanoparticles (CM–NPs) offer design flexibility, enabling functional customization through the selection of membrane sources, while their synthetic cores allow for high drug loading and controlled release [68]. Nevertheless, issues related to batch–to–batch consistency and storage stability remain to be optimized.

**Figure 3:**
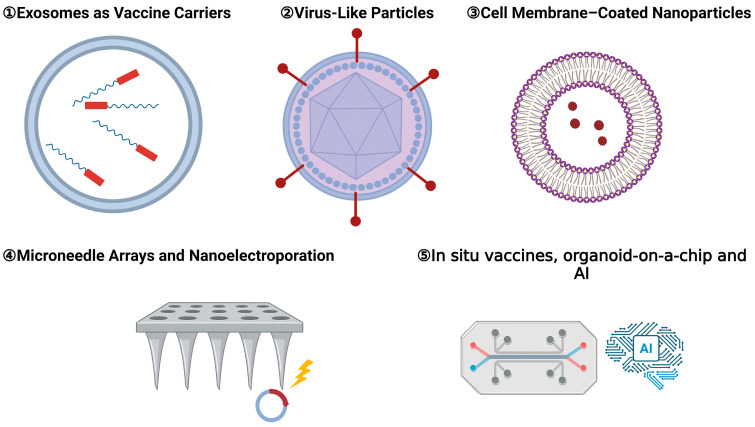
This schematic illustrates five advanced vaccines in biomimetic and cell–derived platforms. ① Exosomes serve as natural nanocarriers that load antigens or adjuvants and target antigen–presenting cells. ② Virus–like particles self–assemble from structural proteins to mimic viruses without infectivity, enabling multiepitope display. ③ Cell membrane coated nanoparticles inherit surface receptors and antigens from source cells for biomimetic delivery and immune modulation. ④ Microneedle arrays with nano–electroporation enable efficient in situ cell transfection and antigen expression in local skin tissue. ⑤ in situ vaccines, organoid–on–a–chip, and AI organoid chips allow high–throughput screening of vaccine efficacy, while AI optimizes antigen design and delivery parameters. Colors are used solely to distinguish different components and carry no additional scientific meaning.

In the realm of clinical translation, lipid nanoparticle (LNP)–based vaccines, exemplified by mRNA–4157, have demonstrated substantial clinical advancements [69]. Concurrently, innovative innate immune adjuvants, including Toll-like receptor (TLR) and stimulator of interferon gene (STING) agonists, are being incorporated into biomimetic delivery systems. Concerning safety considerations, challenges such as immunotoxicity, off–target immune activation, and batch–to–batch variability necessitate meticulous attention during the engineering design phase. Looking forward, the evolution of biomimetic platforms is anticipated to emphasize artificial intelligence–guided neoantigen prediction, modular microfluidic fabrication, and the incorporation of multi–biomarker frameworks. Additionally, the fusion of messenger RNA (mRNA) technology with virus–like particles (VLPs) signifies an innovative trajectory in the development of broad–spectrum vaccines [70,71]. The profound integration of materials science, immunology, and bioengineering is poised to transform the field of next–generation precision vaccines and immunological interventions (Figure 3).

## 4. Physical Delivery Tools

**Figure 4:**
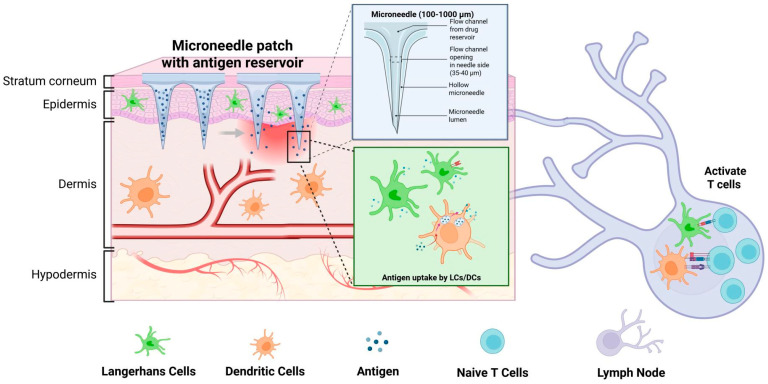
Schematic illustration of microneedle array–mediated cutaneous immunization: MNs penetrate the skin and release antigens; APCs (LCs/dDCs) take up the antigens and become activated; APCs migrate to the lymph nodes; activated T cells generate a systemic immune response. Colors are used solely to distinguish skin layers, microneedle components, and cell types and carry no additional meaning. Arrows indicate the sequential flow of events: antigen release (red arrows), APC migration (green or purple arrows), and T cell activation (blue arrows).

The synthetic nanocarriers and biomimetic platforms described above primarily focus on innovations at the material science level, optimizing antigen presentation by modulating the physicochemical properties of carriers. However, effective antigen delivery depends not only on the carriers themselves but also on the delivery route, the ability to modulate in vivo behavior, and innovations in research and development paradigms. This section will discuss how physical delivery tools (such as microneedle arrays and nano–electroporation) are revolutionizing vaccine delivery from the perspective of administration routes (Figure 4).

### 4.1. Microneedle Arrays

The skin is a highly immunocompetent organ, containing numerous specialized antigen–presenting cells (APCs) such as Langerhans cells (LCs) and dermal dendritic cells (dDCs). These cells mature, migrate to lymph nodes, and present exogenous antigens to CD4^+^ and CD8+ T cells, respectively, to activate subsequent immune responses [72]. Microneedles (MNs) are a novel transdermal drug delivery system composed of a backing and an array of needles, typically ranging in size from 100 to 1000 μm. They create transient microchannels on the skin surface, effectively bypassing the stratum corneum and enabling painless, minimally invasive transdermal delivery [73]. Consequently, intradermal immunization achieved via MNs requires only 1/10 to 1/5 of the traditional intramuscular dose to induce equivalent immune responses, and this enhanced immunogenicity provides a significant dose–sparing effect [74].

Based on their structure and delivery strategies, MNs can be classified into six types: solid, coated, dissolving, hollow, hydrogel–forming, and hybrid designs [75]. Each MN type possesses unique mechanisms, manufacturing techniques, and pharmacokinetic profiles, offering customized solutions for a range of vaccine deliveries. Among these, dissolving microneedles have garnered significant attention in the vaccine field due to their advantages, including no need for removal after use, avoidance of sharps waste, and complete delivery. Fan et al. [76] developed a dissolving microneedle patch employing a spatial segregation strategy, loading influenza split vaccine and RSV pre–fusion F protein into distinct regions of the same patch. By physically separating the antigens, this approach effectively overcomes the common issue of bidirectional antigenic interference associated with traditional combination vaccines, providing proof–of–concept evidence for overcoming antigenic interference in combination vaccine development. Recent studies have further expanded the functional boundaries of microneedles. Nanomaterial–loaded microneedles integrate the aforementioned LNPs, polymeric nanoparticles, and inorganic nanoparticles into microneedle platforms, achieving spatiotemporally controllable co–delivery of antigens and adjuvants [77].

During the COVID–19 pandemic, the stability advantages of microneedle technology were also validated. For instance, the thermostable COVID–19 mRNA microneedle patch (MNP) vaccine developed by Straeten et al. remained stable for up to 6 months at room temperature. This holds the potential to revolutionize vaccine dependence on cold chains, is of great significance for mass immunization in resource–limited regions, and represents a promising platform for the development of next–generation vaccine applications [78].

### 4.2. Nano–Electroporation

Nano–electroporation is a novel physical delivery platform that miniaturizes traditional electroporation technology to the nanometer scale and has demonstrated unique advantages in the vaccine field in recent years.

Based on previous research, electroporation can be categorized into two major directions: ex vivo and in vivo electroporation. The former is a relatively mature technological pathway, referring to the electroporation transfection of isolated cells (e.g., dendritic cells, T cells) ex vivo, followed by the reinfusion of the engineered cells into the body [79]. The advantages of this strategy include high transfection efficiency, controllable conditions, and the avoidance of non–specific effects of electric fields on normal tissues. For example, Chen et al., in a published article, systematically summarized the application of ex vivo electroporation in mRNA–DC vaccines, including the co–transfection of mRNA–encoded TriMix adjuvants (CD70, CD40L, caTLR4) along with antigens into DCs to enhance vaccine immunogenicity [80]. In contrast, in vivo electroporation is performed in situ within living tissues, directly promoting antigen presentation and immune activation. However, its drawback lies in the fact that high voltages can kill a large number of tissue cells and cause significant pain. The emergence of nano–electroporation addresses this issue, as its low–voltage characteristics enable safe in vivo application (e.g., in skin and tumors). Recently, Wang et al. reported a hydrogel–based organic electronic device (μEPO) that combines microneedle electrodes with DNA pre–encapsulation technology. Using a “one–step” operation of in situ electroporation, this device achieves “one–step” delivery and transfection of intradermal nucleic acid vaccines [81]. This in vivo nano–electroporation–based transdermal DNA delivery approach achieved 50% transfection efficiency and a 10–fold dose sparing, demonstrating the strong potential of this strategy in personalized cancer vaccine design and tumor immunotherapy.

## 5. Emerging Strategies

### 5.1. In Situ Vaccine

In situ vaccine (ISV) is an innovative strategy that transforms tumors in situ into “vaccine factories”, activating antigen presentation and anti–tumor immune responses locally within the body (also known as the “abscopal effect”) [82]. The core advantage of this strategy lies in its ability to bypass the need for in vitro identification and synthesis of personalized tumor antigens, instead utilizing the tumor lesion itself as the antigen source, thereby effectively overcoming tumor heterogeneity [83].

The core advantage of this strategy lies in its ability to bypass the need for in vitro identification and synthesis of personalized tumor antigens, instead utilizing the tumor lesion itself as the antigen source, thereby effectively overcoming tumor heterogeneity [84], TLR agonists [85], and physical ablation techniques [86]. Research hotspots and challenges are centered on the optimized modulation of the tumor physical microenvironment, immunosuppressive factors, reduction in antigen loss, and the synergistic application of ablation techniques with immune adjuvants. Concurrently, the introduction of nanotechnology and hydrogel delivery platforms has significantly enhanced antigen presentation efficiency and the persistence of immune activation. For example, Guo et al. encapsulated an oncolytic adenovirus (adv) within a self–assembling peptide hydrogel (Nap gel) and implanted it in situ immediately after tumor resection in a breast cancer mouse model. The sustained–release of the hydrogel enabled continuous release of the oncolytic virus at the surgical site and sustained immune activation, significantly inhibiting postoperative tumor recurrence and metastasis, and inducing immune memory lasting approximately 4 weeks [87]. Leveraging the unique advantages of local delivery, ISV offers an effective pathway that potentially combines potent immune activation with reduced systemic toxicity, holding promise for eliminating both primary and distal/metastatic tumors [88]. We anticipate the rapid development of more effective in situ vaccines for treating different types of tumors, with significant progress expected in various studies currently undergoing clinical translation.

### 5.2. Organoid–on–a–Chip Screening

The study of antigen presentation has long relied on animal models. However, the structural and functional differences between commonly used models like mice and humans, particularly in immune repertoires, cytokine expression profiles, and pathogen recognition pathways, often lead to misleading results, limiting the accuracy of translating findings from animal experiments to human clinical applications [89]. In recent years, the emergence of organoid–on–a–chip (OOC) technology has provided a breakthrough direction to address this challenge. Immune organoids are three–dimensional lymphoid tissue analogs cultured in vitro, capable of simulating key immune processes such as germinal center reactions, B cell antibody class switching, and T–B cell interactions. OOC technology further simulates artificial organs on microfluidic cell culture chips, reconstructing tissue microenvironments and multi–organ interactions [90,91]. For instance, Jeger–Madiot et al. [92] developed a lymphoid organ (LO) chip model. The study demonstrated that myeloid cells within the chip could uptake mRNA delivered by lipid nanoparticles (LNPs), enabling effective evaluation of immune responses to different mRNA vaccine booster strategies. This validates the potential of OOC technology in predicting the immunogenicity of candidate vaccines in humans. A recent study developed a 3D co–culture chip model of pancreatic cancer organoids and T cells (the InterOMaX model), which can be used to assess T cell–mediated tumor killing effects, providing a functional evaluation platform for vaccine screening [93].

In summary, these platforms enable real–time monitoring of dynamic processes such as cell migration, cytokine secretion, and antigen–specific T cell activation, offering high–throughput in vitro models for the screening and optimization of nanocarriers. However, it must be emphasized that current organoids and organ–on–a–chip systems cannot completely replace animal models, they primarily simulate local reactions and struggle to replicate the complete, systemic immune responses present in living organisms. Nevertheless, as complementary tools to animal experiments and pre–screening platforms, they can significantly reduce the number of experimental animals, lower research and development costs, and improve the accuracy of translational predictions.

### 5.3. Artificial Intelligence–Assisted Design

Artificial intelligence (AI), particularly deep learning (DL) technology, is profoundly transforming the paradigm of vaccine development. Traditional antigen/carrier screening relies on large–scale experimental trial and error, whereas AI models can perform high–throughput virtual screening and prediction in silico, shortening work that would take weeks or even months to just hours or days [89].

In the realm of antigen discovery and epitope prediction, structural prediction models like AlphaFold 3 enable precise modeling of protein–antibody complex interactions, providing powerful tools for structure–based antigen design and antibody discovery [94,95]. Some other studies are based on AI models that integrate multi–omics datasets to simulate B cell and T cell responses, cytokine and chemokine signaling, and antigen presentation, offering improved immune–related representations over traditional static models [96]. In nanocarrier design, AI combined with molecular dynamics (MD) simulations can perform both forward prediction and inverse design (recommending optimal carrier parameters based on desired immune response characteristics), enabling the rational design of novel nanodelivery systems [97]. For example, Su et al. used MD simulations to reveal the dynamic conformational changes in ionizable lipids during the organic–to–aqueous phase transition. Simultaneously, using AI–guided screening of candidate lipids, they successfully identified the P1 lipid with a three–tailed conical conformation that promotes the formation of an IgM protein corona, achieving spleen–targeted mRNA delivery. In this study, the P1–mRNA vaccine induced potent antibody and T cell responses and achieved significant tumor suppression in preclinical models [98].

Currently, deep learning and machine learning algorithms are widely applied in epitope prediction, vaccine antigen optimization, nanocarrier design, immune repertoire classification, and the simulation of host–pathogen interactions [99,100]. However, the effectiveness of AI models is highly dependent on the quality and breadth of the training data. The heterogeneity, small sample sizes, and potential biases inherent in current immunological data remain the primary bottlenecks limiting the accuracy of AI–assisted design (Figure 3).

## 6. Conclusions

In this review, three categories of antigen delivery enhancement platforms are examined: synthetic nanocarriers, biomimetic systems, and physical delivery tools. Despite their distinct technical approaches, these platforms share a coherent progression in design philosophy.

Synthetic nanocarriers constitute the foundational tier. They enhance lymph node drainage through precise size control, optimize surface charge to balance stability and cellular uptake, and enable controlled antigen release. Advantages include rational design and scalability. Limitations include heavy reliance on intrinsic physicochemical properties and potential biocompatibility issues. Biomimetic platforms build upon this foundation by leveraging biologically evolved interfaces. They offer superior active targeting, immune modulation, and direct T–cell activation, supported by naturally presented MHC–antigen complexes, repetitive epitope arrays, and preserved membrane protein profiles. Advantages include advanced functionality; limitations include complex preparation, batch variability, and manufacturing challenges. They enable precise spatial delivery to dendritic cell networks and facilitate endosomal escape via physical perturbations. Advantages address delivery efficiency and patient compliance; limitations include device dependence and invasiveness.

When comparing these platforms, they exhibit functional stratification and progressive enhancement. Synthetic carriers provide the groundwork for rational design and production. Biomimetic platforms add advanced targeting and immune modulation. Physical tools solve practical delivery and compliance challenges. These platforms are not mutually exclusive; future optimal vaccine design will likely employ a hybrid strategy integrating a synthetic core, a biomimetic shell, and physical enhancements to achieve comprehensive optimization from molecular design to clinical application.

Despite recent advances, clinical translation faces three main challenges. Firstly, preparation and quality control issues include low yields, batch variability, inconsistent viral particle assembly, and lack of standardized metrics for membrane integrity assessment. Secondly, unpredictable in vivo fate includes protein corona altering targeting, non–specific uptake reducing efficiency, and anti–vector immune responses upon repeated use. Thirdly, validation gap exists as most studies are limited to in vitro or small animal models, lacking direct human evidence for enhanced cross–presentation. Recent studies highlight two key design metrics: epitope density for B–cell activation, and adjuvant subcellular localization for T cell responses [101,102].

Three promising future directions are identified: AI–guided design using machine learning to predict nano–bio interactions; modular and on–demand manufacturing via microfluidic platforms for rapid production of personalized vaccines against emerging diseases or tumor neoantigens [67]; and combination with immune checkpoint inhibitors in tumor vaccines to improve immunotherapy response rates [103]. Achieving clinical success with next–generation antigen delivery tools requires interdisciplinary collaboration among materials science, immunology, bioengineering, and clinical medicine.

## Figures and Tables

**Table 1:** Physicochemical properties and endosomal escape mechanisms.

Nanocarrier	Size (nm)	Key Characteristics	Endosomal Escape Mechanism
LNPs	50–150	pH–responsive charge reversal	Electrostatic interaction disrupts endosomal membrane
Chitosan	150–300	Strong mucosal adhesion	The proton sponge effect leads to increased osmotic pressure and subsequent endosomal rupture
PLGA	100–250	Tunable degradation rate (weeks to months)	Does not directly mediate escape and requires auxiliary strategies
PEI	80–200	High amine group density	A strong proton sponge effect causes endosomal rupture
AuNPs	10–22	Surface plasmon resonance; covalent conjugation capability	Does not rely on endosomal escape and enables direct cytosolic delivery
MSNs	~80	Ultra–high specific surface area; pore size 2–50 nm	Large pores allow physical escape of antigens from lysosomal degradation
IONPs	20–100	Superparamagnetic; biodegradable	Early–late endosomal internalization leads to lysosomal escape within 4 h

**Table 2:** Cross–presentation outcomes and immunological metrics.

Nanocarrier	Cross–Presentation Outcome	Representative Immunological Metrics
LNPs	Enhanced MHC–I cross–presentation	95.9% inhibition rate in the B16–OVA model [9]
Chitosan	Promotes MHC–I cross–presentation	Activates both humoral and cellular immunity [14,15]
PLGA	Dependent on the internalization pathway and often requires auxiliary escape strategies	100% prevention in a lung cancer model and 70% in a melanoma model [16]
PEI	Enables efficient cytosolic delivery and significantly enhances cross–presentation	Strongly enhances OVA–specific T cell responses [17]
AuNPs	Directly presents antigens via MHC–I while bypassing the lysosomal degradation pathway	Induces strong CD8+ T cell responses [18]
MSNs	Exhibits pore–size–dependent cross–presentation efficiency	Large–pore MSNs achieve the highest MHC–I cross–presentation efficiency [19]
IONPs	Possesses intrinsic adjuvant activity and activates the ISG pathway	Generates a CD8+ T cell response that is 55–fold higher than that induced by soluble antigen [20]

## Data Availability

The data are available in the articles included in this review.

## Abbreviations

APC	Antigen–Presenting Cell
MHC	Major Histocompatibility Complex
LNP	Lipid Nanoparticle
DOPE	1,2–dioleoyl–sn–glycero–3–phosphoethanolamine
DSPC	1,2–distearoyl–sn–glycero–3–phosphocholine
DOPC	1,2–dioleoyl–sn–glycero–3–phosphocholine
PEG	Polyethylene Glycol
PLGA	Poly(lactic–co–glycolic acid)
CpG	CpG oligodeoxynucleotide
PEI	Polyethyleneimine
OVA	Ovalbumin
FCS	Fluorocarbon–modified Chitosan
AuNP	Gold Nanoparticle
MSN	Mesoporous Silica Nanoparticle
IONP	Iron Oxide Nanoparticle
MRI	Magnetic Resonance Imaging
ISG	Interferon–Stimulated Gene
STING	Stimulator of Interferon Genes
NOX2	NADPH Oxidase 2
EV	Extracellular Vesicle
ESCRT	Endosomal Sorting Complexes Required for Transport
ILV	Intraluminal Vesicle
MVB	Multivesicular Body
DAMP	Damage–Associated Molecular Pattern
PAMP	Pathogen–Associated Molecular Pattern
TLR	Toll-like Receptor
HIV	Human Immunodeficiency Virus
VLP	Virus-like Particle
DC	Dendritic Cell
BCR	B Cell Receptor
HPV	Human Papillomavirus
SARS–CoV–2	Severe Acute Respiratory Syndrome Coronavirus 2
CM–NP	Cell Membrane–Coated Nanoparticle
TEM	Transmission Electron Microscopy
aAPC	Artificial Antigen–Presenting Cell
CAR–T	Chimeric Antigen Receptor T Cell
EPR	Enhanced Permeability and Retention
LC	Langerhans Cell
MN	Microneedle
RSV	Respiratory Syncytial Virus
MNP	Microneedle Patch
μEPO	Micro–electroporation
ISV	In Situ Vaccine
OOC	Organoid–on–a–Chip
AI	Artificial Intelligence
DL	Deep Learning
MD	Molecular Dynamics
mRNA	Messenger Ribonucleic Acid
siRNA	Small Interfering Ribonucleic Acid
DNA	Deoxyribonucleic Acid
